# Gait Phase Recognition for Lower-Limb Exoskeleton with Only Joint Angular Sensors

**DOI:** 10.3390/s16101579

**Published:** 2016-09-27

**Authors:** Du-Xin Liu, Xinyu Wu, Wenbin Du, Can Wang, Tiantian Xu

**Affiliations:** 1 Guangdong Provincial Key Laboratory of Robotics and Intelligent System, Shenzhen Institutes of Advanced Technology, Chinese Academy of Sciences, Shenzhen 518055, China; dx.liu@siat.ac.cn (D.-X.L.); wb.du@siat.ac.cn (W.D.); can.wang@siat.ac.cn (C.W.); tt.xu@siat.ac.cn (T.X.); 2 Chinese Academy of Sciences (CAS) Key Laboratory of Human-Machine Intelligence-Synergy Systems, Shenzhen 518055, China; 3 University of Chinese Academy of Sciences, Beijing 100049, China; 4 Department of Mechanical and Automation Engineering, The Chinese University of Hong Kong, Hong Kong, China

**Keywords:** gait phase classification, gait phase recognition, lower-limb exoskeleton

## Abstract

Gait phase is widely used for gait trajectory generation, gait control and gait evaluation on lower-limb exoskeletons. So far, a variety of methods have been developed to identify the gait phase for lower-limb exoskeletons. Angular sensors on lower-limb exoskeletons are essential for joint closed-loop controlling; however, other types of sensors, such as plantar pressure, attitude or inertial measurement unit, are not indispensable.Therefore, to make full use of existing sensors, we propose a novel gait phase recognition method for lower-limb exoskeletons using only joint angular sensors. The method consists of two procedures. Firstly, the gait deviation distances during walking are calculated and classified by Fisher’s linear discriminant method, and one gait cycle is divided into eight gait phases. The validity of the classification results is also verified based on large gait samples. Secondly, we build a gait phase recognition model based on multilayer perceptron and train it with the phase-labeled gait data. The experimental result of cross-validation shows that the model has a 94.45% average correct rate of set (CRS) and an 87.22% average correct rate of phase (CRP) on the testing set, and it can predict the gait phase accurately. The novel method avoids installing additional sensors on the exoskeleton or human body and simplifies the sensory system of the lower-limb exoskeleton.

## 1. Introduction

The lower-limb exoskeleton, as a mechanical device that is designed around the shape and the function of the human body and can be worn by the operator [1,2], is widely used for the disabled and elderly people for power-assisted walking or for normal people for load-carrying [3,4]. As a strong coupled humachine system, the walking gait of the lower-limb exoskeleton robot is highly consistent with the human’s [5]. In human walking, the lower-limb joints have similar motion in the same gait phase [6,7]. The walking performance of the exoskeleton is mainly determined by the following three aspects: gait trajectory generation, gait execution and gait assessment [3,8,9], which are all related to gait phases. For example, (1) especially in rehabilitation, the exoskeleton walking gait trajectory is usually generated by a motion model or algorithm based on gait phases [10,11]. (2) Due to the similarity of the motion parameters in the same gait phase, many control strategies of the exoskeleton are developed based on gait phases [12,13]. (3) Extracting the gait phase features and making the segmental gait evaluation are general gait analysis methods [14]. Therefore, accurate gait phase recognition is critical to the lower-limb exoskeleton.

Gait phase is initially described in clinical gait analysis, together with other walking parameters, to diagnose pathological gaits or to evaluate walking after rehabilitation [15]. Human walking is a repetitive lower-limb movement [12]. One gait cycle mainly consists of two states with one leg referenced, the stance phase and the swing phase, and also two states with both legs referenced, single stance and double stances. Each phase is composed of different sub-phases. At present, the mainstream terminology of Rancho Los Amigos (RLA) [16,17] is in use, which has become increasingly popular since the late 1980s. It describes the gait with one leg referenced and includes the stance phase and swing phase. The stance phase consists of the following sub-phases: initial contact, loading response, mid-stance, terminal stance and pre-swing. The swing phase consists of the following sub-phases: initial swing, mid-swing, and terminal swing. This is also a typical classification method [18]. So far, several partitioning models, with different levels of granularity, have been proposed depending on the different clinical aims [19].

In the field of exoskeleton robots, one walking cycle is usually further categorized into multiple phases. S. Oh [20] thought that a single control method is not the best option for all of the motion phases during the gait cycle. H. Kazerooni [21] adopted a hybrid control strategy for different gait phases to control the Berkeley Lower Extremity Exoskeleton (BLEEX). In [21], the walking gait cycle was divided into stance phase and swing phase. Correspondingly, two controllers were developed, which included a position controller used for the stance leg and sensitivity amplification control used for the swing leg. To facilitate the recovery of walking following stroke, S. A. Murray [22] proposed an assistive approach without dictating the spatiotemporal nature of joint movement for the lower-limb exoskeleton. A finite state machine consisting of six gait phase states was used to govern the exoskeleton controller.

In addition to the control of the exoskeleton, the gait phase information is also widely used in gait generation and evaluation. To generate the limb trajectory of the gait robotic trainer, P. Wang [11] marked five gait phase events for one side in one walking cycle. Based on these gait phase events, in total, ten key points were picked up for one gait cycle. T. T. Huu [23] quantified the walking parameters during each gait phase to evaluate the fuzzy-based control strategy on a powered lower-limb exoskeleton. To assess the foot trajectory, Y. Qi [24] adopted the wireless ultrasonic sensor network to detect the human gait phase and to obtain the accurate estimates for the gait cycle, stance phase, swing phase and other gait events.

Seeing the wide application of the gait phase information of the lower-limb exoskeleton, accurate gait phase recognition is the priority among exoskeleton technologies. J. Jung [25] proposed a neural network-based gait phase classification method using sensors equipped on the lower-limb exoskeleton. The classifiers used the orientation of each lower-limb segment and the angular velocities of the joints to output the current gait phase, which was the stance phase or swing phase. In [26], eight different gait phases were separated by using electromyographic (EMG) signal data of the lower-limb to drive foot-knee exoskeleton orthosis. I. Pappas [27] presented a reliable gait phase detection system, which can detect in real time the following gait phases: stance, heel-off, swing and heel-strike. The system employed a gyroscope to measure the angular velocity of the foot and three force-sensitive resistors to assess the forces exerted by the foot on the shoe sole during walking. S. Mohammed [28] adopted an in-shoe pressure mapping system to identify the gait phases. The expectation maximization (EM) algorithm and hidden Markov model (HMM) model are used for dividing one gait cycle into six sub-phases. The work in [29,30] focused on the gait partitioning by means of gyroscope data in order to implement a control system for a pediatric exoskeleton. They introduced a novel algorithm based on a hierarchical weighted decision on the output of two or more scalar HMMs to estimate the most likely sequence of four gait phases. In [31], a gait phase detection algorithm based on HMM, which used data from foot-mounted single-axis gyroscopes, can generate equivalent results as a reference signal provided by force-sensitive resistors (FSRs) for typically developing children and children with hemiplegia. A hybrid method based on a feed-forward neural network (FNN) embedded in an HMM was introduced in [32] for detecting five gait phases. The work in [33] has shown higher performance for HMM than support vector machine (SVM), Gaussian mixture model (GMM) and linear discriminant analysis (LDA) in motion recognition.Therefore, the HMM has a strong ability in modeling the time series action compared to other machine learning methods. However, the neural networks method is good at classifying the actions by the spatial data. The work in [34] proposed that the synchronization between the robots of a team was achieved by exploiting the paradigm of mirror neurons.

In addition, some external assistive approaches for classifying the gait phases are adopted. The paper [35] adopted Phtron FASTCAM Viewer software (FASTCAM-ultima 1024, Photron, Tokyo, Japan) to analyze all video images frame by frame by two research staff members and divided all gait cycles into eight sub-phases independently. In fact, this work took two staff member two months’ time. In [24], three force plates were used to acquire ground reaction forces while walking. The robot data and the ground reaction forces are combined to classify the gait phases. This method is effective, but the hardware cost is higher.

Based on the existing research results, we can get that the gait phase recognition generally used single-type sensors or a combination of multiple types of sensors [18], such as angular velocity, attitude, force, electromyography (EMG), IMU, camera, and so on [24,25,26,27,28,30,36,37,38]. However, for a lower-limb exoskeleton system, as few as possible sensors should be used to achieve the control goal, which can simplify the sensory system and enhance the stability of the exoskeleton. The above sensors used in existing methods have to be additionally installed on the exoskeleton or human, which increase the complexity of the sensory system.

Angular sensors, which are essential for joint closed-loop controlling [39], are usually installed on the joints of the lower-limb exoskeleton. However, other types of sensors, such as force sensors, plantar pressure sensors, attitude sensors or ultrasonic sensors, need to be installed additionally. Actually, the angular sensors of joints already contain the position, velocity, acceleration and other motion information of the exoskeleton robot. Therefore, the detailed gait phases can be recognized accurately using only joint angular sensors based on a reasonable method. However, the derivations of joint rotation velocity and acceleration are definitely affected by accumulated errors from the noise level.

In this paper, we propose a novel gait phase recognition method using only joint angular sensors of the lower-limb exoskeleton. In order to avoid deriving the velocity and acceleration from joint angles, we define a “posture deviation” to represent the “motion”, and then, the posture deviation is used for representing the gait phase features. The method consists of two procedures. Firstly, according to the gait data characteristics, one gait cycle is divided into eight phases by Fisher’s linear discrimination method. To verify the rationality, the effective statistical analysis of the classification results during different walking speeds is also presented. Secondly, the gait phase recognition model based on multilayer perceptron (MLP) neural networks is built and trained by the gait phase-labeled gait set. The experimental results demonstrate that the gait phase recognition model can accurately predict the gait phase label for the lower-limb exoskeleton gait. Therefore, the proposed method can make full use of the potential functions of angular sensors, which are essential for joint closed-loop controlling, to recognize the gait phase. The novel method avoids installing additional sensors on the exoskeleton or human body and simplifies the sensory system of the wearable exoskeleton.

## 2. Materials and Methods

### 2.1. Gait Phase Classification Method with Posture Deviations

Before gait phase recognition, the gait phases of the gait data are firstly classified offline.

According to the analysis of human walking videos and the related literature [6,16,40,41], the hip and knee joints have a smaller motion range while the leg is in the stance phase, and have a larger motion range while in the swing phase. Therefore, when using only joint angular data, it is easier to distinguish different phases within the swing phase than within the stance phase. In general gait phase classifications, the swing phase is usually divided into 3 subphases, including initial swing, middle swing and terminal swing. In the same gait phase, the joints have similar motion features. Here, we also divide the swing phase into the same 3 subphases. However, while the leg switches from swing phase to stance phase, we then take the contralateral leg as a reference and also divide the swing phase into 3 subphases. The transition phase from the left to right swing phase or from the right to left swing phase, namely both feet touching ground, is defined as the double stance phase. Therefore, one gait cycle in this paper is expect to be divided into 8 phases as follows:

- initial left swing phase (*init-LSw*)
- middle left swing phase (*mid-LSw*)
- terminal left swing phase (*term-LSw*)
- double stance phase (*DSt1*)
- initial right swing phase (*init-RSw*)
- middle right swing phase (*mid-RSw*)
- terminal right swing phase (*term-RSw*)
- double stance phase (*DSt2*)

In traditional gait cycle division, one gait cycle is defined as the time from heel-strike to the ipsilateral heel-strike [6]. Then, according to the change of limb orientation and plantar pressure, the gait phases are classified. However, we will introduce a new classification method for gait phase classification with posture deviations (PD) using only joint angular sensors.

#### 2.1.1. Gait Cycle Division Based on Posture Deviations of the Lower-Limb from Standard Standing

In daily life, the natural stance is the most comfortable posture for humans during standing or walking and also has minimal energy consumption. The natural stance is usually completed after minor adjustment based on standard standing, namely standing with feet together. In walking, the lower-limbs deviate from standard standing repetitively [12,37]. For these reasons, we innovatively define one gait cycle as follows: the posture with the minimal lower-limb deviations from standard standing is at the beginning of the gait cycle, and the same posture that appears again is as the end.

**Figure 1:**
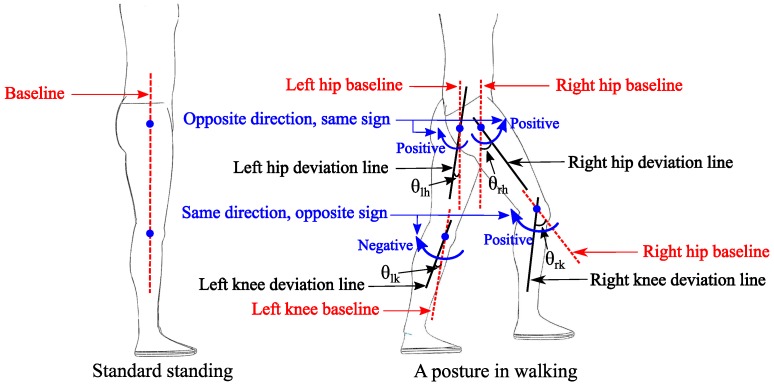
The definitions of the joint angle deviating from standard standing. The left is standard standing, and the right is a posture in walking. The red lines are the baselines, and the black lines are the deviation lines from the baselines. To distinguish the legs, the joint angles are defined as opposite signs, while they are in the same rotary position.

In this work, since we only use the joint angular sensors, accurately, we can only get the ”state” or ”posture” of the lower-limb exoskeleton at a certain moment. Therefore, we define a ”posture deviation” to represent the ”motion”. How does one calculate the posture deviations of the lower-limb from standard standing? We can get the four-joint gait data of lower-limbs, including left hip (notated as lh) flexion/extension, right hip (rh) flexion/extension, left knee (lk) flexion/extension and right knee (rk) flexion/extension. To distinguish the left and right legs, the joint angles are defined as opposite signs while they are in the same rotary position; for example, the left hip flexion is defined as a negative angle; however, the right hip flexion is defined as a positive angle. As shown in Figure 1, the four joint angles that deviated from standard standing in walking are respectively notated as $\theta_{lh}$, $\theta_{rh}$, $\theta_{lk}$, $\theta_{rk}$. A scalar $y_{pd}$ is also defined to describe the posture deviation degree of the lower-limb during walking:

(1) $$y_{pd}=\sqrt{\sum_{i}{\left(\theta_{i}-\theta_{iss}\right)}^{2}},\;i=\left\{lh,rh,lk,rk\right\}$$

where $\theta_{iss}$ is the joint angles of the standard standing posture, which are all defined as $0^{\circ }$. Therefore, Equation (1) can be simplified as:

$$y_{pd}=\sqrt{\sum_{i}\theta_{i}^{2}},\;i=\left\{lh,rh,lk,rk\right\}$$

By calculating $y_{pd}$, the lower-limb deviation degree of the human can be obtained at each time during walking.

#### 2.1.2. Phase Feature Extraction from the Gait Cycle

Human walking is the result of the coupling motion of several lower-limb joints. Additionally, in walking, the angular velocities of each joint change repetitively in one gait cycle [35]. In the initial and terminal swing phases, the hip and knee joints of the swing leg are at higher speeds, so that the foot is away from the ground or close to the ground. However, in the middle swing phase and the double stance phase, the knee joint is at a lower speed, which is almost zero, and the hip joint is also at a lower speed. The gait data, which are 4-dimensional time series, is recorded in angular space, and in fact, they include the joint velocity information. Therefore, with equal interval sampling, the collected gait angular values are some of the more dense or more sparse points along the time axis in Euclidean space where each coordinate axis corresponds to the 1-dimensional joint angular data. Namely, the angular values that have similar densities in Euclidean space along time axis are classified as one phase.

The density information of angular values can be obtained by calculating the Euclidean distance between the adjacent sampling points along the time axis, which is also calculating the posture deviation degree between the current posture and the previous posture. Firstly, we notate G as the gait set including all gait cycles. Each gait cycle is denoted as $x_{i}$, $x_{i}\in G$, i=1,2,…,N, where *N* is the number of total gait cycles. $x_{i}$ is a 4-dimensional gait cycle where *S* is the sampling length of one gait cycle.

(2) $$x_{i}=\left(\theta_{1},\;\theta_{2},\;\ldots ,\;\theta_{S}\right)$$

where:

(3) $$\theta_{j}={\left(\theta_{lh},\;\theta_{rh},\;\theta_{lk},\;\theta_{rk}\right)}^{T},\;j=1,2,\ldots ,S$$

Then, we calculate the deviation distance between the current posture, also the *j* sampling point, and the last posture, also the (j−1) sampling point, and notate $\Delta \theta_{j}$:

(4) $$\Delta \theta_{j}=\sqrt{{\left(\theta_{j}-\theta_{j-1}\right)}^{T}\left(\theta_{j}-\theta_{j-1}\right)}$$

While j=1, let j−1=S; that is, the time sequence is considered as a loop sequence. All of the deviation distances $\Delta \theta_{j}$ of two adjacent postures from the *i*-th gait cycle constitute the *i*-th gait deviation distance vector ${\Delta x}_{i}$:

(5) $${\Delta x}_{i}=\left(\Delta \theta_{1},\;\Delta \theta_{2},\;\ldots ,\;\Delta \theta_{S}\right)$$

Obviously, the angular deviation distance is also considered as the modulus algorithm on angular velocity, where the angular velocities can be approximately obtained by differentiating the angular sequence along the time axis. Therefore, in a physical sense, the gait phase feature extraction method is based on joint angular velocity information [35].

#### 2.1.3. Gait Phase Classification Using Fisher’s Linear Discriminant

Due to the initial and terminal swing phases having higher joint velocities, the middle phase and double stance phase have lower joint velocities, and due to the symmetry of the left and right swing phases, the deviation distances set can be divided into two classes. The larger deviation distances include *init-LSw*, *term-LSw*, *init-RSw* and *term-RSw*; the smaller deviation distances include *mid-LSw*, *DSt1*, *mid-RSw* and *DSt2*.

To divide the deviation distances more accurately, Fisher’s linear discriminant [42] method is adopted. The goal of Fisher’s linear discriminant classification is to make the distances between different classes as far as possible and the distances in the same class as close as possible. The deviation distance sequence is a 2-dimensional sequence including time information. We notate them for the *i*-th gait cycle as a form of set $D_{i}$:

(6) $$D_{i}=\left\{\left(t_{1},h_{1}\right),\left(t_{2},h_{2},\right),\ldots ,\left(t_{S},h_{S}\right)\right\},\;h_{j}=\Delta \theta_{j}$$

where the $t_{j}$ is the time point corresponding to the $\theta_{j}$.

In gait phase classification, the deviation distances set is divided into two classes, namely all subsets of $D_{i}$ are projected to a line and then divided into two classes by Fisher’s linear discriminant method.

According to the characteristics of the walking gait and the deviation distance distribution, the projected line $w_{p}$ is determined as:

(7) $$w_{p}:t=0$$

That means the time factor is not considered, and this also avoids the impact of changing the walking speed. Therefore, the multi-dimensional gait data classification problem becomes a 1-dimensional classification problem.

We denote the two class labels as classI and classII and define a threshold variable *Q*; the classified subset labels of $D_{i}$ is $C=(c_{1},\;c2,\;\ldots ,\;c_{s})$ and:

(8) $$c_{j}=\left\{\begin{array}{c} class\;I,\;\;h_{j}>=Q\\ class\;II,\;h_{j}<Q \end{array}\right.$$

and the mean values of two classes are:

(9) $$\begin{array}{cc} & \mu_{I}=\frac{1}{m_{I}}\sum_{h_{j}\in classI}h_{j}\\ & \mu_{II}=\frac{1}{m_{II}}\sum_{h_{j}\in classII}h_{j} \end{array}$$

where $h_{j}$ is the subset of $D_{i}$, $m_{I}$, $m_{II}$ are the subset numbers of two classes and $S=m_{I}+m_{II}$. The population mean of the set is:

(10) $$\mu =\frac{1}{S}\sum_{h_{j}\in D_{i}}h_{j}$$

According to the scatter definitions for within class and between classes, we get [43]:

(11) $$S_{w}=\sum_{k=I}^{II}\sum_{h_{j}\in classk}\left(\mu_{k}-h_{j}\right){\left(\mu_{k}-h_{j}\right)}^{T}$$

(12) $$S_{b}=\sum_{k=I}^{II}m_{k}\left(\mu_{k}-\mu \right){\left(\mu_{k}-\mu \right)}^{T}$$

The simplified evaluation function with $w_{p}$ : *t* = 0 is defined as [43]:

(13) $$J\left(w_{p}\right)=\frac{S_{b}}{S_{w}}$$

By Fisher’s linear discriminant method, we should minimize the $S_{w}$ and maximize the $S_{b}$. Therefore, we can constantly adjust the *Q* to reclassify the gait set and maximize the function J(·). Correspondingly, the optimal classification threshold $Q_{c}$ is [43]:

(14) $$Q_{c}=arg\;\max_{Q}\;J\left(w_{p}\right)$$

The optimal classification process is as follows:

1. By Equations (8)–(14), we can calculate the optimal classification threshold *Q* for the two fixed classes.
2. According to *Q*, the gait set is classified as two new classes again.
3. Repeat steps 1 and 2 above, until the *Q* is stable, which is also the optimal classification threshold $Q_{c}$.

After determining the threshold $Q_{c}$, the set $D_{i}$ can be divided into *K* regions along the time axis. These are also the new gait phases, and in each phase, their deviation distances are the same or similar.

Additionally, the new classification method is able to dynamically adjust the classification threshold *Q* according to the changing of the gait paces. Meanwhile, the projected line $w_{p}$ : *t* = 0 without considering the time factor determines that this method is not affected by the changing of walking speeds.

### 2.2. Gait Phase Recognition Model

The above is an offline gait analysis method, which can divide one complete gait cycle into multiple phases. In order to be applied to the lower-limb exoskeleton robot, a gait phase recognition model based on MLP is proposed, which can detect in real time the gait phases.

**Figure 2:**
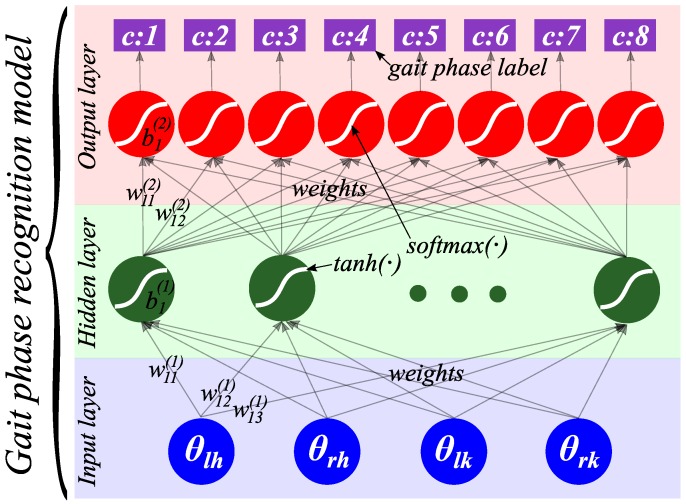
The gait phase recognition model based on multilayer perceptron. The input layer includes a 4-dimensional vector, which corresponds to the four joints from the lower-limb exoskeleton. The middle layer consists of one hidden layer with several hidden units, and the activation function of the hidden unit is tanh(·). The output layer includes eight output units, which corresponds to eight gait phases of one cycle. The function of the output unit is softmax(·). c:1,c:2,…,c:8 are the labels of the gait phases.

With the phase-classified gait set, we build the gait phase recognition model based on multilayer perceptron (MLP) [43] to recognize the gait phase. The MLP is a feedforward neural network, which has a preferable effect for nonlinear classifications. Structurally, as shown in Figure 2, the MLP consists of an input layer, an output layer and a middle layer with one or multiple hidden units. In the gait phase recognition model, we adopt the one-hidden-layer MLP, and the model is:

(15) $$f:R^{I}\to R^{K}$$

where *I* is the size of input vector $\theta_{j}$, I=4, and *K* is the size of output vector f(x), namely the number of classified gait phases, K=8. Shown in Figure 2, the f(x) is [43]:

(16) $$h\left(x\right)=s\left(b^{\left(1\right)}+W^{\left(1\right)}\theta_{j}\right)$$

(17) $$f\left(x\right)=G(b^{\left(2\right)}+W^{\left(2\right)}h\left(x\right))$$

where h(x) is the output vector of the hidden layer, the function G(·) is softmax(·), which is used for multi-class classification, and the activation function s(·) is tanh(·), which typically yields to faster training. $b^{\left(1\right)}$, $b^{\left(2\right)}$ are bias vectors, and $W^{\left(1\right)}$, $W^{\left(2\right)}$ are weight matrices. $W^{\left(1\right)}\in R^{I\times I_{h}}$ is the weight matrix connecting the input vector to the hidden layer, and the $W^{\left(2\right)}\in R^{I_{h}\times K}$ is the weight matrix connecting the hidden layer to the output layer.

In the gait phase recognition model, the parameters set $\beta =\{W^{\left(1\right)},W^{\left(2\right)},b^{\left(1\right)},b^{\left(2\right)}\}$ is initialized randomly. Additionally, stochastic gradient descent [44] with mini-batches is used to learn all parameters of the model based on the classified gait set.

To evaluate the recognition performance of the model, we define the correct rate of set (CRS) and the correct rate of phase (CRP) to quantify the recognition results.

(18) $$CRS=\frac{n_{s\cdot correct}}{N_{s}}$$

where $N_{s}$ is the number of total sample points in the training or testing set and $n_{s\cdot correct}$ is the number of sample points for which the phases are correctly identified in the training or testing set.

(19) $$CRP=\frac{n_{p\cdot correct}}{N_{p}}$$

where $N_{p}$ is the number of sample points in a single phase and $n_{p\cdot correct}$ is the number of sample points that are correctly classified in this phase.

The CRS is used for describing the overall recognition performance of the model during the training or testing process, and the CRP is used for describing the single-phase recognition performance of the model. Through improving CRS gradually, the gait phase recognition model is able to obtain an optimal recognition effect, and then, the recognition effect in each phase of the model can be analyzed by CRP.

### 2.3. SIAT Lower-Limb Exoskeleton Robot

**Figure 3:**
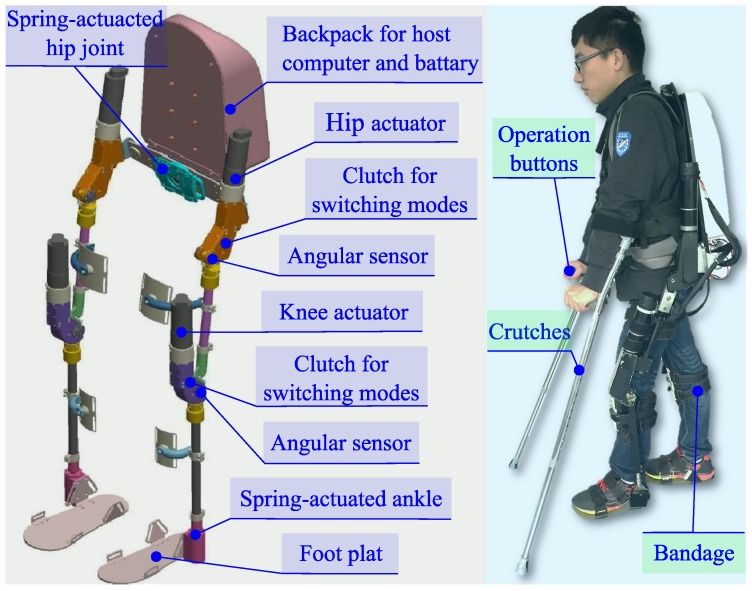
The Shenzhen Institutes of Advanced Technology (SIAT) lower-limb exoskeleton robot. The left is the mechanical structure diagram, and the right shows the exoskeleton worn by the human.

As shown in Figure 3, we have developed a lower-limb exoskeleton robot, named the SIAT exoskeleton, at Shenzhen Institutes of Advanced Technology, Chinese Academy of Sciences, which is mainly used for power-assisted and rehabilitation walking.

Similar to the human lower-limbs, the SIAT exoskeleton has hip joints, knee joints and ankle joints, totaling eight joint degrees of freedom (DoFs). In normal walking, hip abduction/adduction (A/A) is for moving the center of body gravity in the lateral direction, and the flexion/extension (F/E) of hip, knee and ankle is mainly for moving the body in fore-and-aft directions. Therefore, hip A/A, hip F/E, knee F/E and ankle F/E are four indispensable DoFs per leg for normal walking. The distributions of DoFs and actuator types of joints are shown in Table 1. Additionally, the lengths of the thighs and shanks of exoskeleton are adjustable to fit the different heights of the subjects.

The SIAT exoskeleton can work in two modes, power-assisted mode and zero-torque mode, and the clutches are designed on each joint for switching modes. In this work, the gait data are from the SIAT exoskeleton in zero-torque mode, in which the wearers can walk following their own will. Encoders are installed on the actuated joints, including left hip F/E, right hip F/E, left knee F/E and right knee F/E, for recording the walking data. The joint angle sensing specifications are shown in Table 2.

### 2.4. Gait Data Collection

To build a gait phase recognition model over different walking patterns, 20 subjects who have never suffered gait dysfunctions were recruited to participate in this experiment. They were asked to walk on level ground wearing the SIAT exoskeleton in zero-torque mode, and the gait data were recorded by the host computer. As can be known through the relevant literature, the age and height of subjects can more or less affect the lengths of the gait phases [35]. In order to verify the feasibility of our proposed method and also avoid other factors, we narrowed the recruitment range of the subjects. The selected 20 subjects are all males, ages 26 ± 3 years old, with heights of 175 ± 3 cm.

At the beginning of wearing the exoskeleton, the researchers adjusted the length of the exoskeleton legs according to the subject’s shank and thigh, which ensures that the joint axes of the exoskeleton and subject are aligned. Then, the subject performs a random movement to find a comfortable wearing pattern. In the gait data collection, each subject wearing the SIAT exoskeleton walks three minutes with his normal walking patterns and also adjusts the walking speed naturally. Then, the walking speeds of each gait cycle are measured, and 10 gait cycles for which the walking speeds range from 0.8 to 1.5 m/s are randomly selected from the gait set. The gait cycle division has been introduced in Section 2.4, which is different from the description in [16].

**Figure 4:**
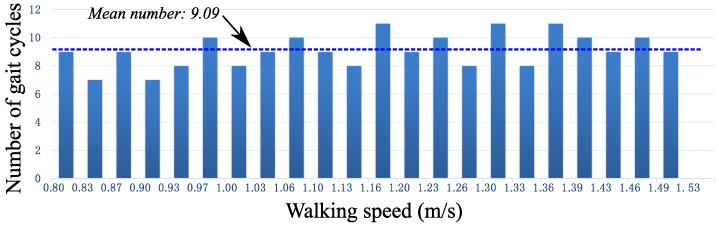
The statistical result of gait cycles from the gait set ordered by walking speed.

Based on the above collection method, a gait set including 200 gait cycles from 20 subjects is established. The statistical result that the numbers of gait cycles fall into different velocity ranges is shown in Figure 4. Obviously, the new gait set is distributed evenly along the walking speed axis, which is convenient to explore whether our new method can correctly identify the gait phase while the walking speed changes.

## 3. Results and Discussion

Above, we present the gait phase classification method and gait phase recognition model using only joint angular sensors, and in this section, the experimental results and discussions will be introduced. Twenty healthy subjects participated in this experiment, and the specifics of the gait data collection have been given in Section 2.4.

### 3.1. Gait Phase Classification Offline

#### 3.1.1. Gait Cycles Division

**Figure 5:**
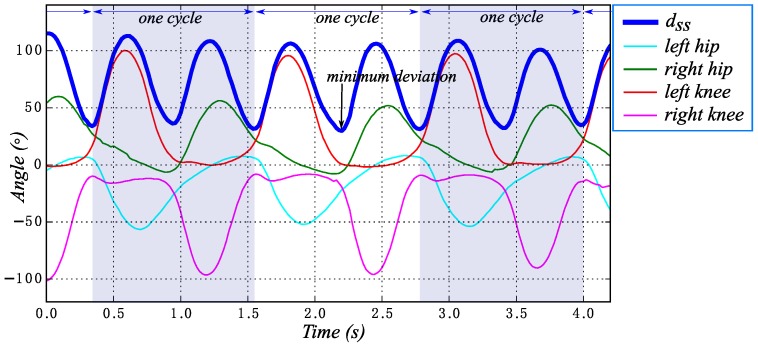
Gait cycle division result with several cycles of one subject walking. The four thin lines below are the original gait. The blue thick line is the deviations curve $y_{pd}$ of the original gait.

Firstly, according to Equation (1), the lower-limb deviations $y_{pd}$ from standard standing in walking are calculated. One walking sequence and the calculation result are given in Figure 5. Obviously, the minimum of $y_{pd}$ is the posture mostly near the standard standing. There are two minimums of $y_{pd}$ in one cycle. By this method, the gait sequence can be automatically divided into several gait cycles. The method is also an optimal choice for exoskeleton gait analysis with only angular sensors.

From the 20 recruited subjects walking with the SIAT exoskeleton, in total, 200 gait cycles are obtained, and they are shown in Figure 4 ordered by the walking speed.

#### 3.1.2. Gait Phase Labels on the Gait Data

After determining the gait cycle, we will verify the classification method.

In Section 3.1.1, 200 gait cycles from 20 healthy subjects have been obtained, and the classification method based on posture deviations is also given in Section 2.1. Therefore, through the above offline gait phase classification method, we divide each gait cycle into multiple phases from 200 gait cycles. The classification results are shown in Table 3. Obviously, 98.5% of the total gait cycles can all be correctly divided into eight phases. The cause of the seven phases is probably the fast limb motion of the swing phase, and the nine phases may be caused by an incoherent walking process. In the following analysis, we mainly use the 98.5% gait cycles with eight phase and define them as effective gait cycles (EGC).

**Figure 6:**
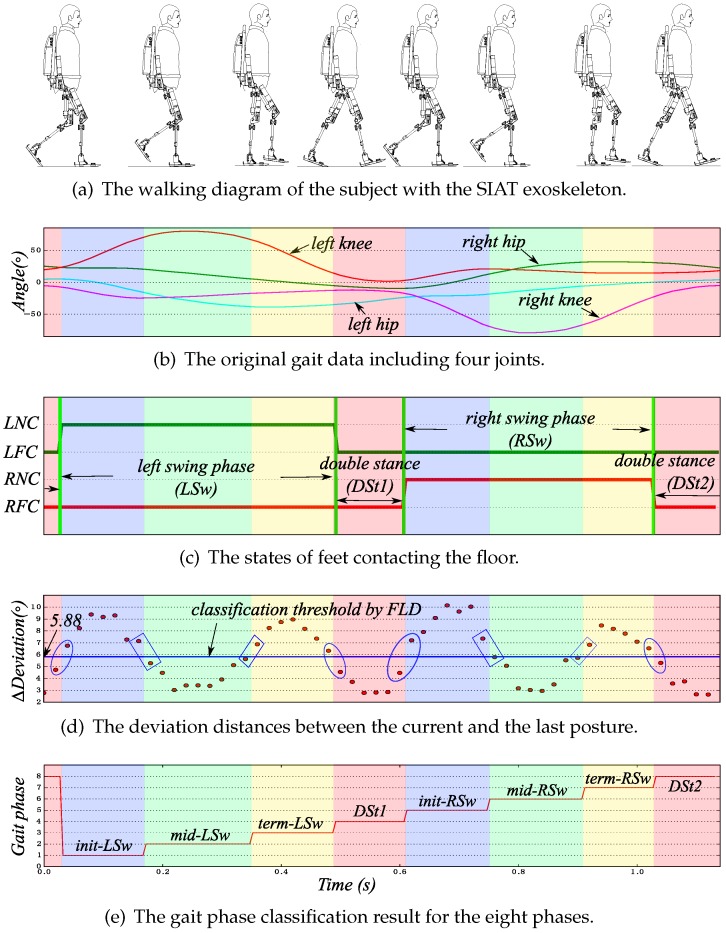
Gait phase classification process of one gait cycle from EGC, where: LNC, left no contact; LFC, left floor contact; RNC, right no contact; RFC, right floor contact.

We take one gait cycle from the EGC into account; the walking diagram with the SIAT exoskeleton, the original gait data, the foot touching the ground, the deviation distances and the gait phase classification results are shown in Figure 6.

As shown in Figure 6a–c, one gait cycle determined by the above method begins approximately from one foot leaving the floor and ends with the ipsilateral foot leaving the floor. However, the separation point of the two gait cycles is not the exact time of the foot leaving the floor. This may be related to different subjects’ walking patterns. Figure 6c presents the state of feet contacting the floor. It is obvious that the classification method is able to accurately recognize the foot touching or leaving the floor.

In Figure 6d, deviation distances are classified by Fisher’s linear discriminant method, and the points in each gait phase are similar or the same. Additionally, the deviation points near the phase boundaries should be further discussed. The calculated deviation distances, which are rounded up by ellipses, are separated easily by Fisher’s linear discriminant method; and those included in rectangles are difficult to separate. This is because, while from the DSt phase to the swing phase or from the swing phase to DSt, the velocities of the swing of the lower-limb joints change rapidly. However, from the initial swing phase to the middle swing phase or from the middle swing phase to the terminal swing phase, the velocities of the swing of the lower-limb joints change slightly.

By the classification results of deviation distances, the gait phase classification results are shown in Figure 6e. The eight phases are *init-LSw* , *mid-LSw*, *term-LSw*, *DSt1*, *init-RSw*, *mid-RSw*, *term-RSw*, and *DSt2*.

**Figure 7:**
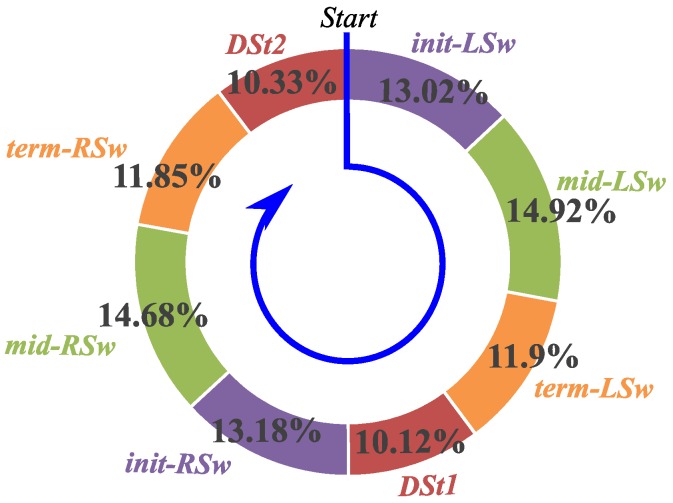
The average percentages of eight gait phases, which are the statistical results of EGC classified by the new gait phase classification method.

The above is the gait phase classification result of one gait cycle from EGC. In order to observe the classification effect of the entire EGC, we perform a statistical analysis on the entire EGC, and the average lengths of each gait phase are shown in Figure 7. Here, the gait phase length is redefined as a percentage of a phase in the cycle.

Generally, with one leg referenced, a gait cycle is divided into two main phases: stance phase and swing phase; and the percentage of the stance phase is 60%; that of the swing phase is 40%. With both legs referenced, a gait cycle is divided into two main phases: single stance and double stance; the percentage of the single stance is 80%; that of the double stance is 20% [6]. The percentage comparisons of the general gait phase classification and the new classification method with posture deviations proposed are shown in Table 4, where single stance includes *init-LSw*, *mid-LSw*, *term-LSw*, *init-RSw*, *mid-RSw* and *term-RSw*, double stance includes *DSt1* and *DSt2* and left swing or right swing includes all *LSw* or *RSw* phases.

According to Table 4, obviously, the gait phase lengths obtained by the classification method with posture deviations are kept consistent with the general method based on the entire EGC, and it also demonstrates that the new gait phase classification method can accurately give the information of foot touching or leaving the ground. As explained above, this is because during the foot touching or leaving the ground, the velocities of the swing of the lower-limb joints change rapidly.

However, Table 4 only gives the division of foot touching or leaving the ground. According to the RLA terminology [41], in the swing phase, the length of the initial swing is 13%; the middle swing is 14%; and the terminal swing is 13%. As shown in Table 5, the three phases corresponds to the initial, middle and terminal swing phases of each leg, where our classification method is referenced by the swing legs. For the left and right initial swing phases, the errors are only 0.02% and 0.18% between the general classification and the new method; however, in the middle phases and the terminal phases, the errors are nearly 1%. This is because, while from the initial swing phase to the middle swing phase or from the middle swing phase to the terminal swing phase, the velocities of the swing of the lower-limb joints change slightly. Therefore, it is difficult to give the swing subphase boundaries accurately, and relatively larger errors have occurred.

From above, the average phase percentages of the classification with PD are nearly consistent with the general classification. That is to say, the classification method using only joint angular sensors has produced effective gait phase classification results. Then, we analyze the gait phase classification results based on the entire EGC and the effects affected by walking speed [35]. Due to the symmetry of legs during walking, the eight gait phases can be divided into left phases and right phases, and the left and right have a one-to-one correspondence. Therefore, the detailed analysis of symmetrical gait phases is as below.

Gait Phases: *init-LSw* and *init-RSw*

**Figure 8:**
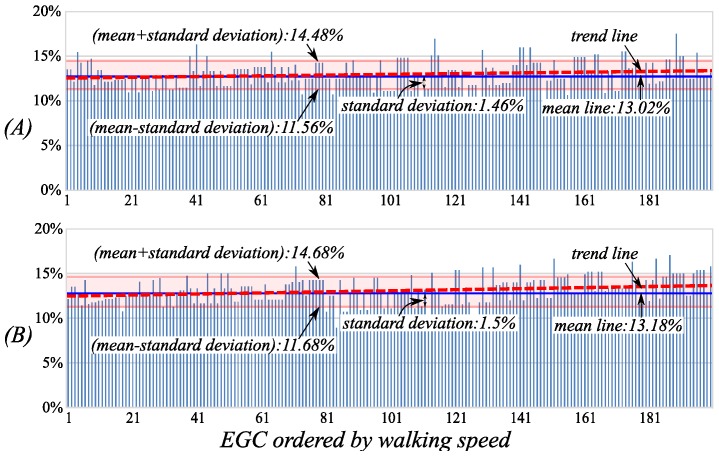
The percentage distributions of the *init-LSw* (**A**) and *init-RSw* (**B**) in the cycle during the EGC. The walking speeds of the EGC are gradually increasing along the horizontal axes.

The percentage distributions of the initial swing phases in the cycle during the EGC are shown in Figure 8. The average values of the two phases are respectively 13.02% and 13.18%, and the errors are only 0.02% and 0.18% relative to the general classification results, 13%, which means the initial swing phases have good separability. From the entire EGC, the two phases have standard deviations of 1.46% and 1.5% respectively, and their trend lines have very small increases with the acceleration of walking speed. This is because the initial swing phases are affected very slightly by the walking speed.

Gait Phases: *mid-LSw* and *mid-RSw*

**Figure 9:**
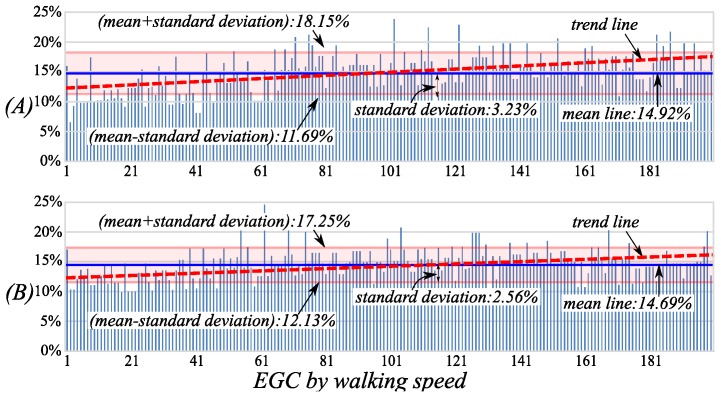
The percentage distributions of the *mid-LSw* (**A**) and *mid-RSw* (**B**) in the cycle during the EGC. The walking speeds of the EGC are gradually increasing along the horizontal axes.

The percentage distributions of two middle swing phases in the cycle during the EGC are shown in Figure 9. The average percentages of the two phases are respectively 14.92% and 14.69%, and they all have bigger standard deviations of 3.23% and 2.56%. It is obvious that the trend lines of the two phases along the walking speed axis are also increasing. That means the percentages of middle phases are more likely affected by walking speed, and the faster the walking speed is, the longer the middle swing phase length is. In addition, the boundaries of the two middle swing phases are difficult to determine due to the slight joint velocities changing at the beginning and the end.

Gait Phases: *term-LSw* and *term-RSw*

**Figure 10:**
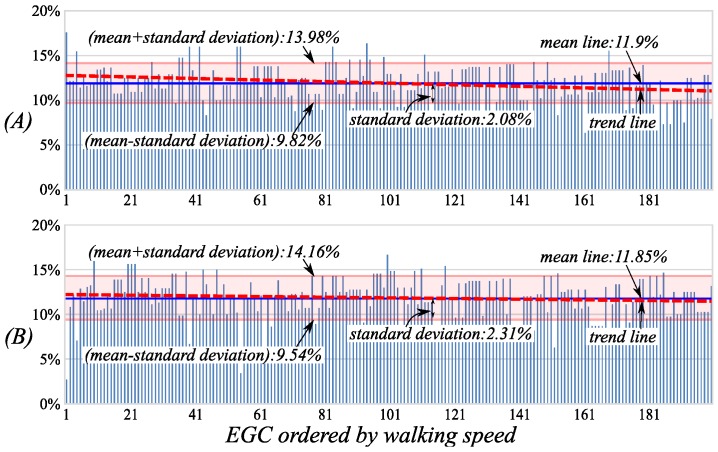
The percentage distributions of the *term-LSw* (**A**) and *term-RSw* (**B**) in the cycle during the EGC. The walking speeds of the EGC increase gradually along the horizontal axes.

The percentage distributions of the two terminal swing phases are shown in Figure 10. The average percentages of the two phases are respectively 11.9% and 11.85%, which also have bigger deviations of 2.08% and 2.31%. However, the trend lines are decreasing slightly during the EGC with the walking speed increasing. This is because the two terminal swing phases are affected slightly by the walking speed, and the instability of the gait phase length during EGC may be caused by the greater changing of the middle swing phases.

Gait Phases: *DSt1* and *DSt2*

**Figure 11:**
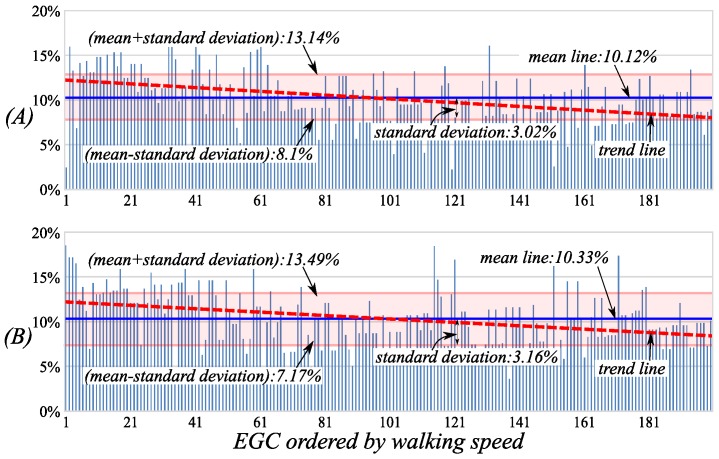
The percentage distributions of the double stance Phase 1 (*DSt1*) (**A**) and *DSt2* (**B**) in the cycle during the EGC. The walking speeds of the EGC are gradually increasing along the horizontal axes.

In the clinical setting, the two phases are usually measured by plantar pressures, which are both 10% of the gait cycle. Additionally, by our classification method, the two phases are respectively 10.12% and 10.33%, and the errors are only 0.12% and 0.33%. By Figure 11, the two phases have bigger deviations of 3.02% and 3.16%, whose trend lines decrease obviously while the walking speeds increase. This is because the double stance phases are affected obviously by the walking speed, and the faster the walking speed, the shorter the double stance phases.

**Figure 12:**
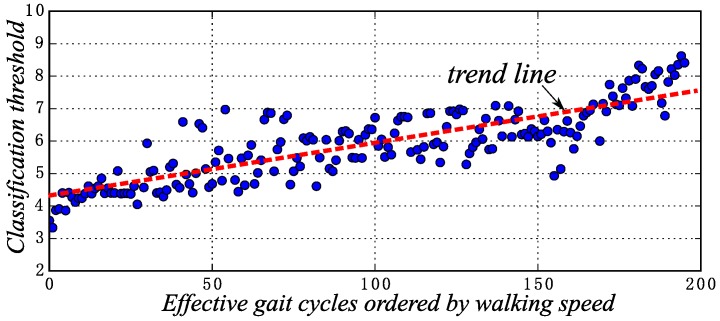
The classification threshold distributions during the EGC. The walking speeds of the EGC increase gradually along the horizontal axis.

The above analysis gives the gait phase classification results and the rationality of gait phases. The gait phases are more or less affected by walking speed, and different phases have different effects. Adopting Fisher’s linear discriminant method, the classification threshold is able to be adjusted dynamically with the walking speed changing, which is shown in Figure 12. With the walking speed being faster, the classification threshold also increases.

So far, one gait cycle can be divided into eight phases correctly by the new gait phase classification method, and the classification threshold can be adjusted dynamically with the changing of walking speeds. Meanwhile, the advantage of the method only uses the joint angular sensors installed on the lower-limb exoskeleton. However, this is just an offline gait phase classification method.

### 3.2. Gait Phase Recognition

In EGC, the 197 gait cycles are from 20 subjects, which include 17 subjects, each of whom has 10 gait cycles, and the remaining three subjects, each of whom has nine gait cycles. In order to evaluate the gait phase recognition model more comprehensively, we adopt cross-validation to verify the model performance. Firstly, the 20 subjects are randomly divided into five groups, and each group includes four subjects. While training the model each time, the group of four subjects is the testing set, and the remaining 16 subjects are the training set. Therefore, the five groups are used one-by-one as the testing set, until the training ends. Here, we have 10,835 sample points in total with gait phase labels, 8768 for training and 2157 for testing. The input vector of the model has only four dimensions; however, the output is an eight-dimensional vector corresponding to eight gait phases. Therefore, the neural network model should have more hidden units to enhance the performance of the classifier. The classification results during different numbers of hidden units are shown in Table 6 and Table 7.

**Figure 13:**
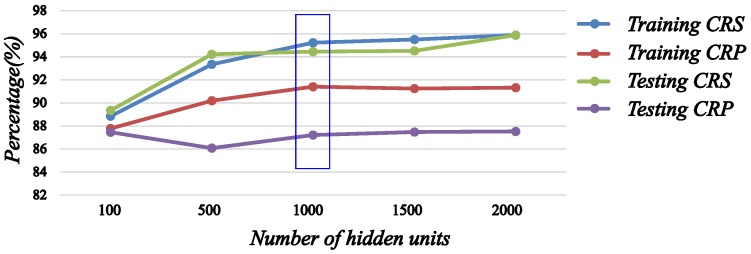
The CRS and CRP of the gait phase recognition model for different numbers of hidden units.

From Table 6, the more hidden units of model there are, the higher the correct rate of recognition on the training set; however, the maximum of the average CRP on the training set is 91.41%, while the number of hidden units is 1000. On the testing set, the CRS and CRP of the model are also increasing with more hidden units, but when the number of hidden units is greater than 1000, the CRS and CRP change very slightly. In order to present the changing of CRS and CRP more clearly for different numbers of hidden units, we show them in Figure 13. Structurally, with the number of hidden units increasing, the computational efficiency of the model will gradually decline, which will affect the real-time application. Therefore, the determination of the number of hidden units not only depends on the recognition rate of the model, but also is limited by the complexity of the model. Through the overall consideration, we determine the number of hidden units as 1000.

**Figure 14:**
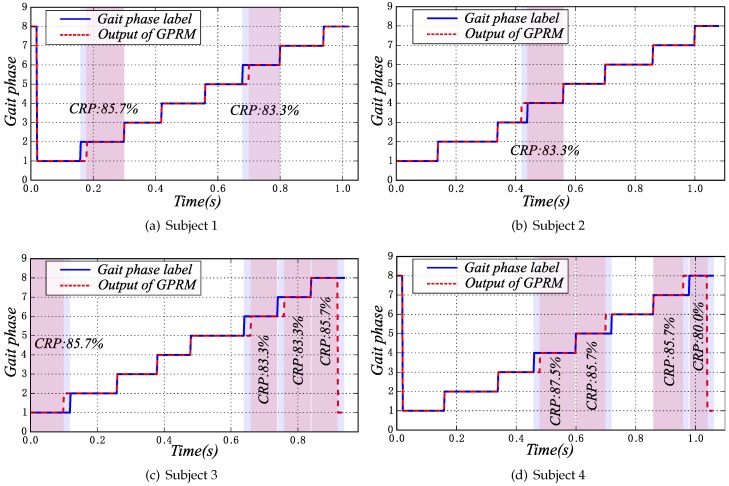
The gait phase recognition results of four cycles, which are respectively from four subjects of the test set. GPRM, gait phase recognition model.

In the cross-validation, each time the gait phase recognition model is trained based on the 16 subjects’ gait data, the remaining four subjects’ data are used for testing the model. Here, the gait phase recognition results of four gait cycles, which are respectively from four subjects of the first test set, are shown in Figure 14.

In Figure 14a, the *mid-LSw* and *mid-RSw* phases have recognition errors; the other phases are all predicted accurately. In Figure 14b, only one gait phase *DSt1* has recognition errors, and in Figure 14c and Figure 14d, there are four gait phase recognition errors respectively. However due to the smaller sampling rate, each gait phase has only a few gait data points. Therefore, once one gait data point is identified by mistake, the correct recognition rate will be affected severely. From Figure 14, the CRPs of the four cycles are all less than 90%. As shown in Table 7, while the number of hidden units is 1000, from the whole gait set, the CRS is 94.45%; however, from the single phase, the CRP is only 87.22%. Further analysis found that, in each of the phases in which appear the identification errors, in fact, only one gait data point is identified by mistake. Therefore, the gait phase recognition model has been able to predict the gait phase labels accurately.

## 4. Conclusions

The gait phase recognition for lower-limb exoskeletons has become a hot research topic due to its extensive application. So far, many approaches to identify the gait phase have been developed; however, additional sensors that the existing methods use need to be installed, such as plantar pressure, attitude or IMU sensors. To make full use of the existing joint angular sensors, which are essential for closed-loop controlling, we proposed a novel gait phase recognition method using only lower-limb joint angular sensors. According to the characteristics of the gait data, we redefined the eight gait phases, which are referenced by the swing legs. To extract the gait phase features, the deviation distances are calculated and classified by Fisher’s linear discriminant method. Then, the gait phase labels of the gait set are obtained. By offline gait data classification, one gait cycle can be correctly divided into eight phases. To verify the rationality of the novel method, the relationships between the length of each gait phase and the walking speed are also analyzed. With the gait phase-labeled data, we build a gait phase recognition model based on the multilayer perceptron neural network. By training the model with a four-dimensional input vector and an eight-dimensional output vector, we can recognize in real time the gait phase through the four lower-limb joint angular data. For the testing set, the model has 94.45% of CRS and 87.22% of CRP. The experimental results demonstrate the effectiveness of the gait phase recognition. The novel method also simplifies the sensory system of the lower-limb exoskeleton.

Above, some research findings have been obtained. However, the CRS and CRP are relatively lower, because of the lesser amount of sampling points. Therefore, in the next work, we will increase the sampling frequency and decrease the gait phase classification and recognition error. Moreover, in future work, we intend to improve the existing control strategy of the lower-limb exoskeleton by the novel gait phase recognition method and to develop the gait trajectory evaluation method for the rehabilitation exoskeleton.

## Figures and Tables

**Table 1:** The joint actuator types. A/A, abduction/adduction; F/E, flexion/extension.

Joint	DoFs	Actuator Type
Hip A/A	2	Spring-actuated
Hip F/E	2	Motor-actuated
Knee F/E	2	Motor-actuated
Ankle F/E	2	Spring-actuated

**Table 2:** Joint angle sensing specifications.

Item	Value
Number of encoders	4
Sampling resolution	10 bit
Angular precision	$0.35^{\circ }$
Frequency of sampling	50 Hz
Data transmission speed	115.2 kbit/s

**Table 3:** The gait phase classification results of 200 gait cycles.

*K* Phases	7 Phases	8 Phases	9 Phases
Number of cycles	1 (0.05%)	197 (98.5%)	2 (0.1%)

**Table 4:** The average main phase percentage comparisons of the general classification and the 3 new method.

Gait Phase	General Classification [6]	New Classification
Single stance	80%	79.55%
Double stance	20%	20.45%
Left stance	60%	60.16%
Left swing	40%	39.84%
Right stance	60%	60.29%
Right swing	40%	39.71%
Left half cycle	50%	49.96%
Right half cycle	50%	50.04%

**Table 5:** The average swing phase percentage comparisons of the general classification and the new method. init, initial; LSw, left swing phase; RSw, right swing phase; term, terminal.

Gait Phase	General Classification [41]	New Classification
*init-LSw*	13%	13.02%
*init-RSw*	13%	13.18%
*mid-LSw*	14%	14.92%
*mid-RSw*	14%	14.68%
*term-LSw*	13%	11.90%
*term-RSw*	13%	11.85%

**Table 6:** The average training set correct rate of set (CRS) and correct rate of phase (CRP) of the gait phase recognition model through 5-fold cross-validation for different numbers of hidden units.

Number of Hidden Units	100	500	1000	1500	2000
CRS	88.85%	93.35%	95.22%	95.51%	95.89%
*init-LSw* CRP	88.75%	91.68%	93.22%	92.66%	93.12%
*mid-LSw* CRP	91.88%	94.32%	95.55%	95.22%	94.95%
*term-LSw* CRP	86.17%	86.22%	87.98%	87.68%	86.90%
*DSt1* CRP	88.86%	91.89%	92.12%	94.05%	93.86%
*init-RSw* CRP	87.68%	91.08%	90.87%	90.76%	91.58%
*mid-RSw* CRP	87.26%	88.64%	90.21%	89.90%	90.71%
*term-RSw* CRP	83.12%	86.23%	87.25%	87.78%	87.50%
*DSt2* CRP	88.50%	91.52%	94.05%	92.02%	92.05%
Average CRP	87.78%	90.20%	91.41%	91.25%	91.33%

**Table 7:** The average testing set CRS and CRP of the gait phase recognition model through 5-fold cross-validation for different numbers of hidden units.

Number of Hidden Units	100	500	1000	1500	2000
CRS	89.35%	94.22%	94.45%	94.52%	95.88%
*init-LSw* CRP	89.05%	87.23%	89.34%	90.86%	92.12%
*mid-LSw* CRP	88.23%	88.05%	89.18%	88.31%	87.36%
*term-LSw* CRP	89.92%	87.29%	89.04%	90.05%	92.08%
*DSt1* CRP	84.16%	85.56%	86.55%	87.22%	88.15%
*init-RSw* CRP	87.50%	86.59%	87.97%	85.98%	85.03%
*mid-RSw* CRP	89.68%	87.93%	87.25%	87.24%	88.92%
*term-RSw* CRP	84.23%	81.85%	83.16%	82.75%	80.21%
*DSt2* CRP	85.92%	84.17%	85.25%	86.36%	86.33%
Average CRP	87.46%	86.08%	87.22%	87.47%	87.53%
